# Research Progress on the Synthesis of Nanostructured Photocatalysts and Their Environmental Applications

**DOI:** 10.3390/nano15090681

**Published:** 2025-04-30

**Authors:** Yanan Niu, Qi Shi, Tai Peng, Xi Cao, Yuguang Lv

**Affiliations:** 1 College of Materials Science and Engineering, Jiamusi University, Jiamusi 154007, China; 2 School of Art and Design, Qiqihar University, Qiqihar 161006, China; 3 School of Information Science and Technology, Beijing University of Chemical Technology, Beijing 100029, China; 4 College of Pharmacy, Jiamusi University, Jiamusi 154007, China

**Keywords:** nanomaterials, photocatalysis, synthesis method, multiple applications, environment

## Abstract

Due to their unique photocatalytic properties, nanostructured photocatalysts have shown broad prospects for application in environmental treatment. In recent years, researchers have significantly enhanced the photocatalytic charge separation efficiency and photocatalytic stability of photocatalysts by regulating semiconductor energy band structures, optimizing interface and surface properties, constructing heterogeneous structures, and introducing noble metal doping. This review systematically summarizes the basic principles, synthesis methods, and modification strategies of nanostructured photocatalysts and focuses on recent research advances in their environmental applications, such as water pollution control, air purification, and carbon dioxide reduction. Meanwhile, this review analyzes current challenges in the field, such as low quantum efficiency, insufficient stability, and limited industrialization, and outlines future development directions, including smart catalytic technology, fabrication of multifunctional composites, and large-scale synthesis, thereby providing a reference for research and application.

## 1. Introduction

With the acceleration of global industrialization, environmental pollution has become a core challenge that limits sustainable development [1,2,3,4,5]. Against this background, photocatalytic technology, which can directly harness solar energy to drive chemical reactions and facilitate pollutant degradation, is regarded as a significant breakthrough in green technology [3]. In recent years, nanostructured photocatalysts have significantly improved light absorption efficiency, charge separation ability, and catalytic activity through strategies such as morphology regulation, bandgap engineering, and surface—interface modification, showing great potential in the environmental field [6].

Photocatalytic technology excites a semiconductor catalyst with light to produce e^−^-h^+^ pairs [7]. The core lies in optimizing the separation and migration efficiency of photogenerated carriers through material design, thereby improving the catalytic reaction rate [8]. Compared with traditional catalytic technology, photocatalysis does not require additional energy input and can directly utilize solar energy. It has unique advantages in the fields of environmental purification and energy conversion [9] and has become an important research direction in the environmental field.

**Figure 1:**
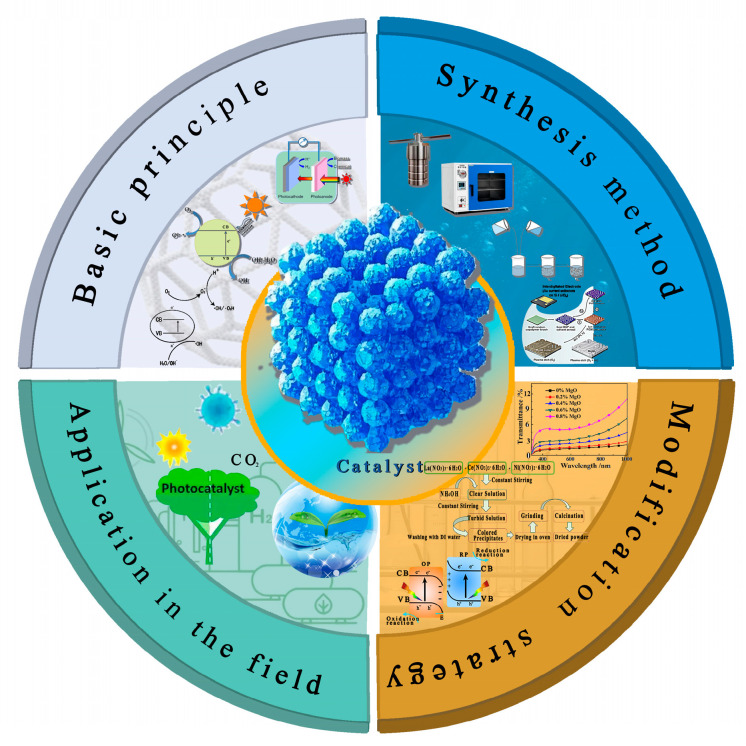
Synthesis and application of nanostructured photocatalysts.

Due to their high specific surface area [10], quantum confinement effect [11], and unique electron transmission characteristics, nanomaterials exhibit obvious advantages in the field of photocatalysis [12,13]. By regulating the size, morphology, crystal structure, and surface chemical properties of the nanomaterials [14], their light absorption characteristics, e^−^-h^+^ separation efficiency [10,15], and catalytic stability can be effectively optimized. For example, graphene, which has excellent light absorption capacity and quantum efficiency [16], has been applied in biosensors and fluorescence imaging sensors [17]; 1D materials (such as CNTs), which exhibit excellent mechanical, electrical, and thermal properties [18], can be used to prepare high-performance composite materials, electronic devices, and hydrogen storage materials [19]; Due to their ultra-thin structures, 2D materials (such as MoS_2_ and g-C_3_N_4_) exhibit the ability of rapid electron migration [20,21], and the 3D nanostructure can provide abundant active sites and relatively short diffusion paths [22]. In addition, the construction of a multi-component composite structure can further improve the catalytic performance [23,24] and expand its application potential [25]. As shown in Figure 1, it presents the synthesis and application of nanostructured photocatalysts, etc.

**Figure 2:**
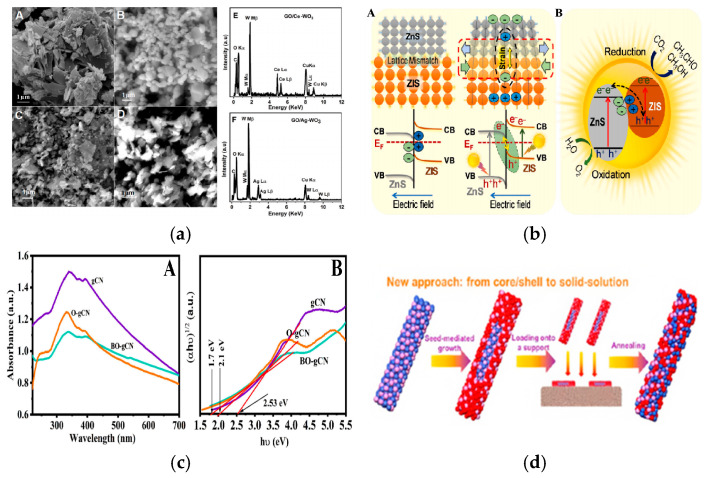
Modification strategy of nanostructured photocatalysts (**a**) SEM images of WO_3_ (**A**), Ag nanoparticles (**B**), GO/Ce-WO_3_ (**C**) GO/Ag-WO_3_ (**D**) nanostructures. EDX mapping of GO/Ce-WO_3_ (**E**) and GO/Ag-WO (**F**). Reproduced from Ref. [10] with permission from Elsevier, copyright 2025. (**b**) Schematic illustration of lattice mismatch and strain effect between cubic ZnS and hexagonal ZIS phases in ZnS/ZIS-3 (**A**) and Direct Z-scheme of ZnS/ZIS (n–n^+^) heterojunction in ZnS/ZIS-3 (**B**) Reproduced from Ref. [30] with permission from Elsevier, copyright 2025. (**c**) UV-Vis spectra for bulk and doped (B and O)-gCN (**A**) and Tauc plot for those samples (**B**). Reproduced from Ref. [31] with permission from Elsevier, copyright 2025. (**d**) New approach to CoMPx Nanorods (NRs) mediated by core/shell nanostructure formation. Reproduced from Ref. [33] with permission from ACS, copyright 2025.

In recent years, the research of nanostructure photoelectric catalysts has made important progress, especially in the modification of semiconductor materials [26], the analysis of catalytic mechanisms [27], and the expansion of applications [28], in which researchers have proposed a variety of innovative strategies. For example, precious metal co-catalysts were introduced [10]. As shown in Figure 2a, SEM images of Ag and Ce-doped WO_3_ and GO nanostructured catalysts are presented to show their microstructures. In addition, a Z-type heterostructure is constructed [29]. As shown in Figure 2b, a Z-type heterojunction of ZnS/ZIS (n^−^n^+^) is constructed in ZnS/ZIS-3, and it contains non-metallic elements [30]. As shown in Figure 2c, the UV-Vis spectrum of the g-C_3_N_4_ material doped with B and O is presented [31,32]. As shown in Figure 2d, the use of the plasma effect is a new synthesis method of core-shell structure nanomaterials with surface plasma. These strategies have effectively improved the stability of photoelectric catalytic materials, the separation efficiency of photogenerated carriers, and the catalytic selectivity of target molecules [33]. In terms of applications, the nanostructured photocatalytic catalysts have been widely used in organic pollutant degradation [34,35], air purification [36,37], carbon dioxide reduction [38,39], water decomposition and hydrogen production [40,41], photoelectric cells [42], and energy storage devices [43], etc. However, the field still faces some challenges, such as low quantum efficiency [44], photocorrosion [45,46], insufficient stability [47], and high industrialization preparation costs [48], etc. Therefore, future research will pay more attention to the development of high-efficiency catalysts, the in-depth understanding of catalytic mechanisms, the introduction of intelligent catalytic technologies, and the feasibility of large-scale applications so as to promote the practical application of photocatalytic technology.

The aim of this review is to systematically analyze the recent research advances in nanostructured photocatalysts, to discuss in depth the strategies for their design, preparation, and performance optimization, and to comprehensively assess the prospects for their application in the environmental and energy fields. Compared with the existing literature, the unique contributions of this review are to focus on the mechanism of microstructure modulation of nanomaterials on photocatalytic performance and summarize a variety of material design strategies; to analyze the applications of photocatalysts in the fields of water pollution treatment and air pollution control in detail; and to critically analyze the major problems in current research and propose solutions and future research directions. Through these efforts, this review aims to provide valuable references and guidance. It also aims to promote the practical application of photocatalytic technology and fill the gap between existing research and practical applications.

## 2. The Basic Principle of Nanostructure Photocatalysts

Nanostructured photocatalysts drive a series of redox reactions through the generation, separation, and migration of photogenerated charge carriers and are widely used in the treatment of environmental pollution. Their performance is affected by factors such as the energy band structure of the material, the light absorption capacity, the kinetic characteristics of the charge carriers, and the activity of the surface catalytic reaction. Therefore, the rational design and optimization of nanostructured photocatalysts are of great significance for improving their catalytic efficiency. This section systematically summarizes the basic principles of photocatalysis.

**Figure 3:**
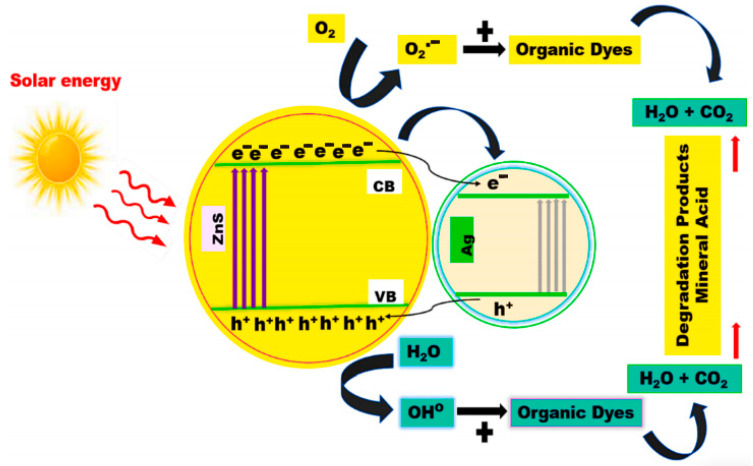
Photocatalytic Mechanism of 1.5 wt% of Ag-doped ZnS nanoparticles under sunlight irradiation. Reproduced from Ref. [51] with permission from Elsevier, copyright 2025.

Nanostructured photocatalysis is a technology that drives chemical reactions through light energy [49]. Its core is to achieve efficient energy conversion and material transformation by utilizing the unique photoelectric characteristics of nanomaterials [33,50]. As shown in Figure 3, the basic principle is based on the core mechanism of photocatalysis: Photocatalysts such as TiO_2_ absorb photons, the valence band electrons jump to the conduction band to form electron-hole pairs, and the combined effect of the built-in electric field and the defect state promotes their separation, and the electrons and holes are subsequently transported in the bulk phase and to the surface to participate in the reaction [51]. In heterojunction photocatalysts, Type-II heterojunctions transfer electrons and holes to the conduction and valence bands of the other semiconductor due to energy band interleaving; the built-in electric field of p-n heterojunctions drives the separation of electrons and holes. Interfacial states can assist charge transfer at the heterojunction interface, and different interfacial contact modes also affect the charge separation efficiency [52]. The separation of these photogenerated e^−^-h^+^ pairs thereby suppresses their recombination and enhances the interfacial reaction kinetics. The quantum-limited domain effect and high specific surface area of nanostructures enable a 2–3-fold increase in carrier generation efficiency and a shift in the migration path from bulk-phase dominated to surface-dominated (shortening of the migration distance to 10–50 nm) [53]. For example, the carrier recombination can be reduced by directional transmission in one-dimensional nanowire structures [54], while the surface adsorption and reaction activity can be enhanced in two-dimensional nanofilms due to the exposure of more edge sites [48].

Energy band engineering is a key strategy for regulating photocatalytic performance. By constructing heterogeneous structures (such as Type-II, Z-type, or Schottky junctions), an internal electric field can be formed to promote the spatial separation of photogenerated charge carriers [55]; defect engineering (such as oxygen vacancies and nitrogen doping) can introduce intermediate energy levels, optimize light absorption, and enhance the adsorption and activation ability of reactants (such as H_2_O and CO_2_) [49,56]. The charge transfer mechanism at the surface interface directly affects the reaction kinetics: after photogenerated carriers migrate to the material surface, electrons can reduce adsorbed heavy metal ions (e.g., Hg^2+^ → Hg^0^) or participate in hydrogen-producing reactions, and holes can oxidize organic pollutants (e.g., mineralize phenol to CO_2_) [57], thereby suppressing photodegradation and improving catalyst stability [58]. In the current research, the in situ spectroscopic techniques [59] (such as in situ Raman and XPS) and theoretical calculations (DFT) [60] have been employed to reveal the microscopic mechanism, such as the reconstruction of the interface double electric layer and the change of the adsorption energy of the intermediate body, thereby providing a theoretical basis for the precise design of efficient photocatalytic catalyst systems [61,62]. In the future, it is necessary to further explore the dynamic synergy effects under multi-field coupling (light, electricity, and heat), thereby promoting its breakthrough in environmental applications.

## 3. Synthesis of Typical Nanostructured Photocatalysts

The preparation method of nanostructured catalysts directly affects their crystal structure, defect concentration, specific surface area, and surface active sites. Therefore, it is necessary to select the optimal preparation process for different catalyst systems. At present, common synthesis methods include the solvent thermal/hydrothermal method, sol-gel method, vapor deposition method, template method, ionothermal treatment, photodeposition method, and electrochemical deposition method. This section selects several synthesis methods for a summary so as to provide ideas for the subsequent preparation of catalysts.

### 3.1. Solvothermal/Hydrothermal Synthesis

**Figure 4:**
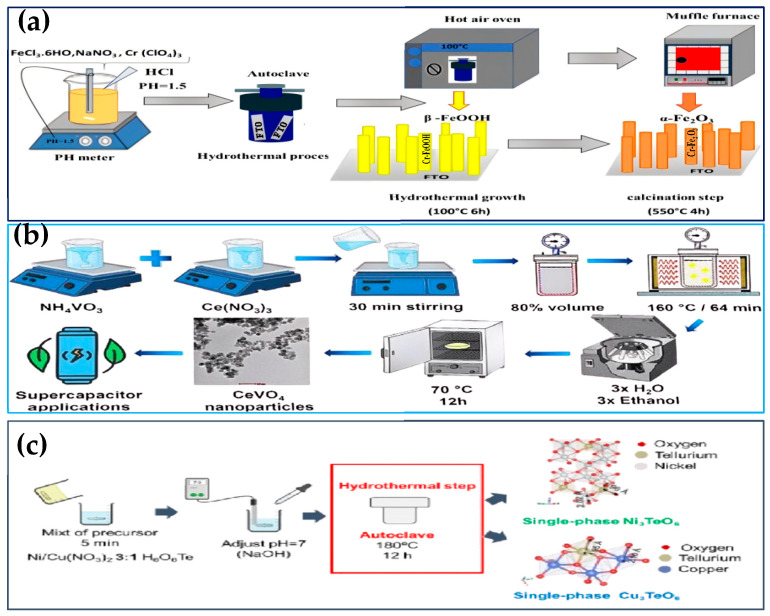
Hydrothermal synthesis of nanostructured photocatalysts. (**a**) Reproduced from Ref. [64] with permission from Springer Nature, copyright 2025; (**b**) Reproduced from Ref. [65] with permission from Elsevier, copyright 2025; (**c**) Reproduced from Ref. [66] with permission from ACS, copyright 2025.

The solvothermal or hydrothermal method is one of the common methods for the synthesis of nanostructured photocatalysts. Using solvents such as water and alcohols in a closed autoclave promotes the crystallization and growth of nanomaterials at high temperatures and pressures. This method is suitable for the synthesis of nanostructured photocatalysts with high crystallinity, uniform particle size, and controllable morphology, such as TiO_2_, BiVO_4_, g-C_3_N_4_, etc. Tiwari et al. [63] used the hydrothermal method to react the mixed solution of iron—zinc nitrate, ammonium fluoride, and urea at 120 °C for 12 h. After that, they washed and dried the product and then fired it at 350 °C for 2 h to synthesize a ZnFe_2_O_4_ film on the foam nickel substrate. The synthesized material exhibits a superior capacitance ratio and long-term cycling stability. Barros et al. [64] used Ce(NO_3_)_3_·6H_2_O and NH_4_VO_3_ as precursors, dissolved and mixed them at 90 °C. Then, they adopted the microwave hydrothermal method (160 °C, 2.5 GHz, 64 min) to synthesize CeVO_4_. The product was washed with water to neutralize the pH and then dried at 80 °C for 12 h (as shown in Figure 4a). This method shows its application potential in supercapacitors. Bouhjar et al. [65] used the hydrothermal method to react the acidic solutions of FeCl_3_, NaNO_3_, and Cr(ClO_4_)_3_ for 6 h at a pH of 1.5 and a temperature of 100 °C. The maximum photocurrent density and incident photon conversion efficiency were achieved when the Cr doping was 16% (as shown in Figure 4b). Fernández et al. [66] prepared Na-free Ni_3_TeO_6_ (37 nm) and Cu_3_TeO_6_ (140 nm) nanocrystals (as shown in Figure 4c) by the hydrothermal method at pH 7. The enhanced antiferromagnetic interactions (TN reached 57 K and 68 K, respectively) have provided new ideas for the applications of magnetoelectric materials and optical detectors. The solvothermal/hydrothermal method grows the precursor crystals in a confined high-temperature and high-pressure environment, which can regulate the nanoparticle size, morphology, and crystal shape, and the resulting photocatalysts have a complete crystal structure, few defects, high corrosion resistance, and stable activity. The nanoparticles can be recovered by centrifugation and filtration, but they are easy to agglomerate, and some of the particles will be adsorbed on the wall of the container or the filtration medium, which will affect the recovery rate. The method requires a large amount of organic solvent, which is costly and environmentally unfriendly, and the reaction time is long. The synthesis can be extended by increasing the number of laboratory reactors, but reactor sealing and heat and mass transfer need to be optimized for actual production. The reaction is reproducible, and strict control of the conditions leads to consistent products with photocatalytic degradation efficiencies exceeding 85%.

### 3.2. Sol-Gel Method

**Figure 5:**
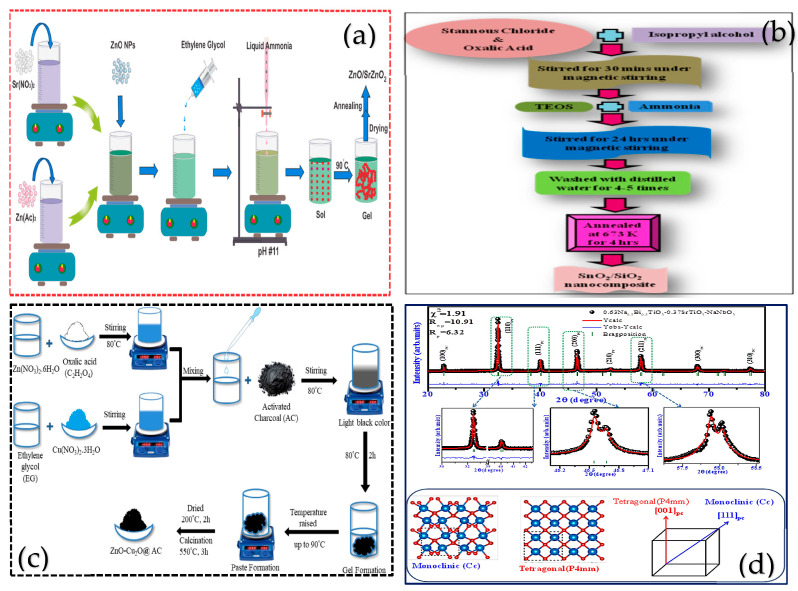
Synthesis of nanostructured photocatalysts by the sol-gel method (**a**,**b**) Reproduced from Refs. [15,67] with permission from Elsevier, copyright 2025; (**c**,**d**) Reproduced from Refs. [71,72] with permission from Springer Nature, copyright 2025.

The sol-gel method, a wet chemical synthesis technology based on the hydrolysis and gelation of metal precursors, is suitable for preparing uniformly dispersed metal oxide catalysts. It enables precise control of the chemical composition and impurity elements and is amenable to large-scale production. Ahmad et al. [67] used Zn(CH_3_COOH)_2_ and Sr(NO_3_)_2_ as precursors to form a soluble gel by adjusting the pH to 11 and then added 0.5 g of ZnO. After gelation at 90 °C, the mixture was calcined at 300 °C for 2 h (Figure 5b). The resulting composite exhibited the potential to mineralize toxic dyes and kill pathogenic microorganisms. Giordano et al. [68] synthesized metal nitrides and metal carbides using the sol-gel method, and their catalytic performance was improved due to the larger surface area of these materials and the easier formation of small particles. Kumar et al. [15] co-precipitated tin dichloride (SnCl_2_), oxalic acid (C_2_H_2_O_4_), ethyl orthosilicate (Si(OEt)_4_)/isopropanol (C_3_H_8_O), and ammonia water, and then stirred the mixture for 24 h. After that, the mixture was fired at 673 K for 4 h to obtain the composite material SnO_2_/SiO_2_ (as shown in Figure 5a). Compared with the single SnO_2_, the composite material exhibited an approximately five-fold increase in surface area and enhanced electro-optical performance. Laghrib et al. [69] synthesized Mn-doped TiO_2_, Sn-doped TiO_2_, and Mn-Sn co-doped TiO_2_ using the sol-gel method. The precursor was titanium tetrakis (2-propanolato). After hydrolysis with acetic acid, gelation occurred, and then MnCl_2_ and SnCl_2_ were added for doping. The mixture was calcined at 500 °C for 2 h to obtain a photocatalytic material with a doping amount of 0.5–1%. The Mn and Sn co-doped TiO_2_ material resulted in a decrease in band gap energy and an increase in the decomposition rate of methylene blue per unit time. Martin Mark et al. [70] used the sol-gel method to prepare Sn-doped (1–5 wt%) NiMn_2_O_4_ nanoparticles. The doping of Sn reduced the band gap of NiMn_2_O_4_ from 2.17 eV to 1.85 eV. Moreover, the 5% doped sample exhibited a specific capacitance of 758 F/g at 1 A/g. Additionally, within 120 min, the photocatalytic degradation rates of MO and RhB were 91% and 97%, respectively. These results demonstrate the potential for the synergistic application of efficient energy storage and photocatalysis. Chitalkar et al. [71] successfully constructed a new ternary ZnO-Cu_2_O@AC photocatalyst. Compared with the binary ZnO-Cu_2_O composite material, its degradation rate of RhB can reach 99.87%, as shown in Figure 5c. This is mainly attributed to the enhanced visible light absorption, rich surface active sites, and efficient e^−^-h^+^ separation. These characteristics have provided a new type of model material for low-cost water remediation. Satyanarayana et al. [72] used acetate as the precursor, which was first gelatinized and then calcined in multiple steps (450 °C/4 h, 900 °C/3 h) to obtain high-purity 0.63Na_0.5_Bi_0.5_TiO_3_-0.37SrTiO_3_-NaNbO_3_ ceramics, as shown in Figure 5d. This process improved the overall energy storage performance of these ceramics. The sol-gel method prepares materials with high homogeneity, can be performed at room temperature and pressure, is chemically stable, is low cost, and has good reproducibility. However, the gel time is long, and the drying and curing are prone to defects. In large-scale production, the uniformity and consistency of the drying and curing process are difficult to control, limiting scalability. The catalyst can be filtered and centrifuged for recovery, but the powdered form is easy to dust and lose, and the surface is easy to adsorb intermediates, or the structure collapses and clogs the pores after many cycles, so that the activity and the recycling efficiency are reduced. Materials synthesized in this way can degrade some organic pollutants by up to 80–95%.

### 3.3. Chemical Vapor Deposition (CVD)

**Figure 6:**
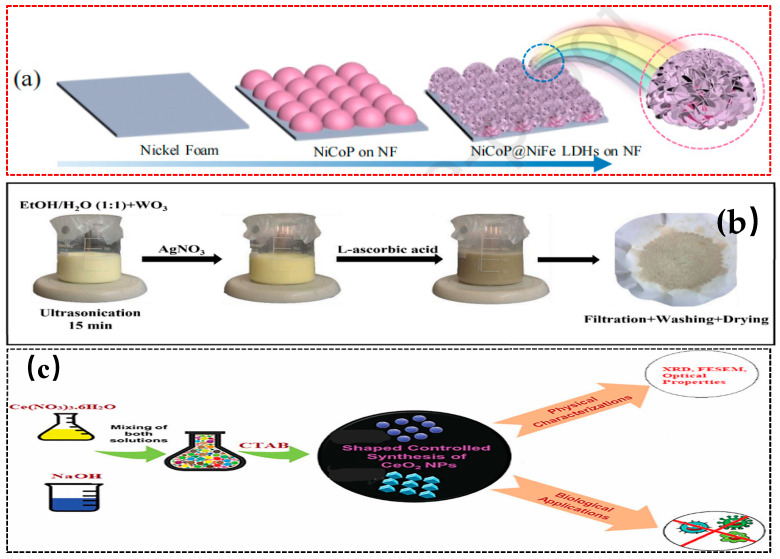
Synthesis of nanostructured photocatalysts by vapor precipitation (**a**) Reproduced from Ref. [74] with permission from Elsevier, copyright 2025; (**b**) Reproduced from Ref. [75] with permission from Elsevier, copyright 2025; (**c**) Reproduced from Ref. [77] with permission from Taylor & Francis, copyright 2025.

Gas-phase deposition methods, including chemical vapor deposition (CVD), atomic layer deposition (ALD), etc., are suitable for preparing thin-film catalysts and are commonly used in the integration of photoelectrochemical devices. Mazhdi et al. [73] used the co-precipitation method to mix Zn(NO_3_)_2_ and Gd(NO_3_)_3_ solutions and then added NaOH dropwise and aged at 80 °C for 2 h. After being washed, dried at 100 °C, and annealed at 250 °C for 2 h, Gd-doped ZnO nanoparticles were obtained, which have good potential as ultraviolet photodetectors, luminescent materials, and optoelectronic materials. Li et al. [74] constructed a CoNiP@NiFe LDHs heterostructure through two-step electrodeposition (as shown in Figure 6a). The interfacial electronic rearrangement optimized the HER adsorption free energy and OER hole enrichment, demonstrating efficient water-splitting performance. Matalkeh et al. [75] synthesized Ag/WO_3_ using a deposition-precipitation method. They ultrasonically dispersed WO_3_ in a water/ethanol mixture, sequentially added CTAB, AgNO_3_, and ascorbic acid, and then stirred to deposit Ag nanoparticles. After washing and drying, the mixture was calcined at 300 °C for 1 h to obtain the Ag/WO_3_ composite material (as shown in Figure 6b). The Ag particles are uniformly dispersed on the surface of WO_3_, and the bandgap of the composite material is reduced from 2.6 eV to 2.1 eV. Under visible light irradiation, the degradation rate of MB reaches 80% within 120 min. Moreover, the composite material exhibits dose-dependent antibacterial activity against both Gram-positive and Gram-negative bacteria, combining efficient catalysis with antibacterial stability. Arshad et al. [76] utilized chemical vapor deposition to achieve the controlled growth of carbon nanofibers on vertically aligned indium arsenide nanowires. This method employs vertical InAs nanowires that are prepared by molecular beam epitaxy on an Au/InAs (111) B substrate. After annealing to form Au nanoparticles, TMIn and TBAs were used as precursors for a 45-min growth process at 430 °C. The controlled growth process of carbon nanofibers on InAs nanowires provides a novel heterogeneous integration strategy for constructing high-performance materials. Sebastiammal et al. [77] used CTAB as a template agent to co-precipitate Ce(NO_3_)_3_·6H_2_O with NaOH at 60 °C, followed by calcination at 600 °C for 3 h. The green method involved mixing sweet basil leaf extract with Ce(NO_3_)_3_·6H_2_O, evaporating at 80 °C, and then calcining at 600 °C for 2 h. Both methods produced pure-phase C-CeO_2_ and G-CeO_2_, as shown in Figure 6c. These materials exhibited antibacterial activity against Staphylococcus aureus and Escherichia coli. This indicates their potential to cause bacterial cell death. Alsaedi et al. [78] used a precipitation process, employing nitrate solutions with different Zn/Lu weight ratios, adjusted the pH to 7.2 to generate carbonate precipitates, and after washing by centrifugation, drying at 120 °C for 3 h, and calcining at 750 °C for 2 h, they obtained ZnO-Lu_2_O_3_ nanocomposites. The study found that the ZnO-Lu_2_O_3_ nanocomposites have higher catalytic activity than pure ZnO NPs. The vapor phase deposition method can accurately control the thickness and morphology of the material to prepare high-quality vapor phase deposition method can accurately control the thickness and morphology of the material to prepare high-quality films with fast reaction speed and better stability of the synthesized material. However, the reaction conditions are harsh, the equipment is complicated and costly, and it is difficult to produce on a large scale, and the quality of the product varies from batch to batch. If the photocatalyst is deposited on a specific substrate, the recovery is easy and the loss is small; if the nanoparticles are deposited in the gas phase, specialized equipment such as an electrostatic precipitator and cyclone separator is needed to recover them, and the process is complicated. However, the composites synthesized by this method can increase the degradation rate by 30–50%.

### 3.4. Template-Assisted Synthesis

**Figure 7:**
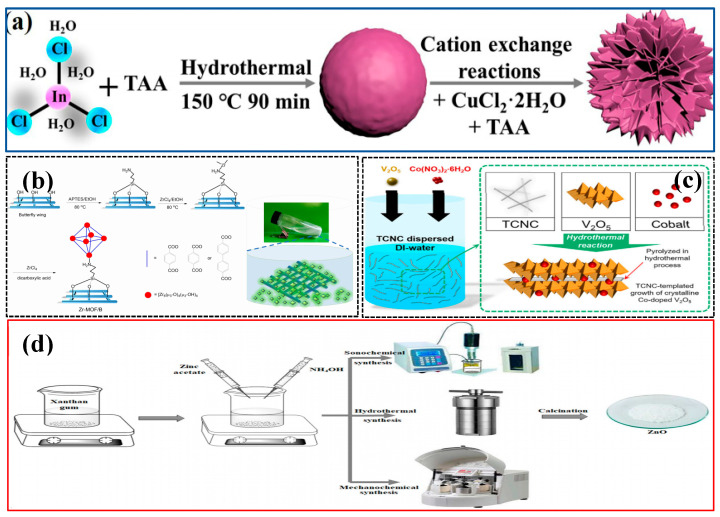
Synthesis of nanostructured photocatalyst by template method (**a**) Reproduced from Ref. [81] with permission from MDPI, copyright 2025; (**b**) Reproduced from [79] with permission from Chemistry Europe, copyright 2025; (**c**) Reproduced from [83] with permission from Elsevier, copyright 2025; (**d**) Reproduced from Ref. [82] with permission from Springer Nature, copyright 2025.

The template method uses hard templates (such as SiO_2_ nanospheres) or soft templates (such as surfactants) to guide the growth of nanomaterials and is often used to synthesize catalysts with porous structures. Singh et al. [49] reported on the preparation of Pt/IrO_2_ nanocatalysts using PS-b-PVP block copolymer templates. By enhancing the loading capacity through pyridyl alkylation, they found that the mass activity of nanocylinders improved by 35–94% compared to layered materials, revealing the critical impact of mesostructure on catalytic performance. They also developed a high-throughput electrochemical evaluation platform based on an interdigitated electrode chip and provided new strategies for the study of the relationship between nanostructures and properties. Sun et al. [79] anchored UiO series Zr-MOF films on the surface of butterfly wing photonic crystals to construct oriented nano-composite photoelectrodes (as shown in Figure 7b). The synergistic effect of MOFs and biological photonic crystals significantly enhances the photoelectrocatalytic hydrogen evolution performance, with the cathodic current density reaching up to 3.64 mA/cm^2^, providing a biomimetic composite strategy for the design of efficient solar-driven electrodes. Huo et al. [80] used a Bi_2_Te_3_ template to synthesize two-dimensional PtBiTe hexagonal nanosheets, which exhibited superior methanol oxidation activity (3.1 times higher specific activity) and stability in alkaline media compared to commercial Pt/C. In situ infrared and theoretical calculations confirmed its CO-free catalytic pathway, providing a new strategy for the design of efficient fuel cell catalysts. Liao et al. [81] constructed a sulfur vacancy-containing In_2_S_3_/CuInS_2_ heterojunction microflower photocatalyst (as shown in Figure 7a) through an In_2_S_3_ microsphere template-guided metal ion exchange strategy. Under visible light, the CO and CH_4_ production rates reached 80.3 and 11.8 μmol g^−1^ h^−1^, respectively, which are 4 times and 6.8 times higher than pure In_2_S_3_. This enhancement is attributed to the heterojunction promoting charge separation, the microflower structure enhancing light absorption, and the sulfur vacancies suppressing recombination, thus providing a new approach for the on-demand design of efficient metal sulfide photocatalysts. Kaur et al. [82] synthesized highly crystalline and high surface area ZnO nanostructures using xanthan gum as a biological template in combination with sonochemistry. The catalyst prepared by the template-assisted method exhibited excellent photocatalytic performance under ultraviolet light, with a degradation rate of 99.6% for triclosan and 96.09% for imidacloprid, due to its low band gap and high porosity. This provides a green synthesis strategy for efficient degradation of emerging pollutants. Youn et al. [83] synthesized cobalt single-atom catalysts (CoVO NBs) anchored on V_2_O_5_·nH_2_O nanobands using a template method (as shown in Figure 7c). The Co sites in the catalyst can regulate the binding energy of reaction intermediates, exhibiting high stability in the oxygen evolution reaction (OER) (overpotentials of 428/374 mV in 0.1/1 M KOH) and long-cycle performance in zinc-air batteries (450 h). This provides a new strategy for addressing the aggregation and support corrosion issues of single-atom catalysts. The template method can accurately control the morphology and size of the photocatalyst, but its cost is high, the removal of the template is complicated and easy to destroy the structure, the residual template will occupy the active site and affect the stability, the reproducibility is general, and the complex preparation process will also limit the scalability. Complex pore structure is prone to adsorption of large molecule contaminants and clogging of the pore to reduce stability. The recovery method depends on the catalyst morphology: regular pore structure catalysts can be recovered by filtration and centrifugation, but it is necessary to avoid operation damage to the pore structure; the recovery of loaded catalysts needs to consider the interaction between the carrier and the catalyst and to prevent dislodging from affecting the recovery and reuse. The specific surface area of the material synthesized by this method is 2–3 times higher than that of conventional catalysts, which significantly enhances the adsorption and degradation of pollutants.

### 3.5. Other Methods

In addition to the above methods, there are several other methods for synthesizing nanostructured photocatalysts. For example, Pelicano et al. [84] reviewed the synthetic strategies, structural characterization, and performance optimization progress of metal poly (heptazinamide) (MPHI) photocatalysts. They especially emphasized the key role of ionothermal synthesis methods in the preparation of MPHI with high crystallinity. The review pointed out that MPHI has high activity in photocatalytic hydrogen production, hydrogen peroxide production, and carbon dioxide reduction for solar energy fuel production. Additionally, it explored the challenges faced in this research field. Goto et al. [85] prepared the Al-doped SrTiO_3_ photocatalyst by the ionothermal synthesis method. This photocatalyst has an apparent quantum efficiency of 56% at 365 nm, can rapidly release gases in a 1 mm aqueous layer without the need for forced convection, and achieves a solar hydrogen production efficiency of 0.4% in 1 m^2^ of light-accepting area. These characteristics provide a feasible method for large-scale photocatalytic hydrogen production. Manukumar et al. [86] prepared one-dimensional mixed-phase TiO_2_ nanorods using an ionothermal synthesis method. These nanorods exhibited higher hydrogen generation activity than P-25 under solar light irradiation, attributed to the unique electron-hole complexation mechanism as well as the efficient separation and transfer of photogenerated carriers in the one-dimensional mixed-phase TiO_2_ system. This study reveals the potential of the ionothermal synthesis method for the preparation of highly efficient photocatalysts and highlights its promising application in the field of photocatalytic hydrogen production. Xing et al. [87] synthesized NiO/KNbO_3_ nanocomposites by photodeposition for photocatalytic nitrogen fixation. The method achieves uniform loading of NiO on the surface of KNbO_3_ under mild conditions, and the optimal sample ammonia production rate reaches 470.6 mmol g^−1^ h^−1^, which is 4.8 times higher than that of pure KNbO_3_, demonstrating highly efficient photocatalytic performance. The detailed comparison of these synthesis methods is summarized in Table 1.

In the study of nanostructured photocatalysts, the establishment of a comprehensive multi-dimensional and multi-scale characterization system is crucial for stability evaluation. Cyclic stability testing is one of the core assessment tools. In conducting such tests, 5 to 10 repetitions of photocatalytic experiments, such as dye degradation or hydrogen production reactions, need to be carried out under fixed light source intensities, reactant concentrations, and temperatures, which, in combination with reaction kinetic analysis, can clarify the deactivation mechanism of the catalyst [88]. Structural stability is monitored by using XRD to detect crystalline phase transitions (e.g., the transformation of TiO_2_ from anatase to rutile), combining with TEM/SEM to observe morphological degradation, and applying nitrogen adsorption-desorption (BET) to verify changes in the specific surface area (where the decrease should be less than 20%) [89]. In terms of chemical stability, Inductively Coupled Plasma Emission Spectroscopy (ICP-OES) accurately detects the concentration of dissolved metal ions (e.g., the dissolution of Ti^3+^ should be less than 1 ppm). Meanwhile, XPS resolves the evolution of the chemical state of surface elements (e.g., the reduction in metal oxides induced by photocorrosion) [90]. Photostability requires tracking the shift of the light absorption edge by UV-Vis DRS and monitoring the change in the carrier complexation rate by PL. In summary, the synergistic application of these evaluation metrics helps deeply understand the stability of photocatalysts, guides the optimization of synthesis strategies, breaks through the bottleneck of poor stability, and promotes the further development of nanostructured photocatalysts in practical applications [91].

**Table 1:** Synthesis methods, brief principles, etc., of nanostructured photocatalysts.

**Synthesis Method**	**Brief Principle**	Precursor/Raw Material	Reaction Conditions	Name of Nanomaterials	Ref.
Solution Combustion Method, SCM	The high-temperature and high-pressure environment promotes the combination of zinc ions with oxygen	Metal salts, reducing agents, water	Atmospheric pressure combustion	ZnO nanoparticles	[92]
Hydrothermal Method	Fe_3_^+^-β-FeOOH-α-Fe_2_O_3_-Cr_3_^+^ replace	Metal salts, deionized water, FTO conductive glass, etc.	Acidic conditions, high-temperature hydrothermal, and annealing	Cr-doped hematite, α-Fe_2_O_3_:Cr	[66]
Hydrothermal Method	The high temperature and high pressure in the hydrothermal environment control the growth of crystals	Metal salts, deionized water, NaOH, etc.	Alkaline, Constant temperature is maintained inside the autoclave	Strontium Titanate Nanocubes, STNCs	[93]
Binder-free hydrothermal method	Nanomaterials are formed in a high-temperature and high-pressure hydrothermal environment	Metal salts, CO (NH_2_)_2_ etc.	A sealed environment for high-temperature and high-pressure autoclaves	Binder-free ZnFe_2_O_4_ nanosheets on nickel foam	[63]
Sol-Gel Technique	The p-n heterojunction promotes the effective separation of e^−^-h^+^ pairs and enhances their activity	Metal salts, C_2_H_6_O_2_, deionized water, etc.	Alkaline, high-temperature calcination	Nanostructured ZnO/SrZnO_2_ Composite	[67]
Sol-Gel Technique	Hydrolysis generates metal complexes, which are then heated and calcined to prepare the materials	Metal salts, EG, deionized water, etc.	Alkaline high-temperature heating calcination	Ternary ZnO-Cu₂O@AC Nanocomposite	[71]
Sol-gel Method	Band gap regulation and Charge separation enhancement	Metal salts, deionized water, etc.	stir, gel drying, High-temperature calcination	Mn-Sr co-doped TiO_2_	[69]
Sol-gel Method	Lattice doping and structure regulation	Metal salts, NH_3_·H_2_O, etc.	Alkaline, gel drying, high-temperature calcination	Sn:NiMn_2_O_4_	[70]
Sol-gel Method	The hydrolysis and polycondensation reactions of the precursor form a sol, which is then dried and calcined	SnCl_2_, NH_3_·H_2_O, etc.	Mix and stir, filter, and calcine	SnO_2_/SiO_2_	[15]
CVD	Construction of heterogeneous structures	WO_3_, AgNO_3_, deionized water, etc.	Ultrasonic, stirring, drying, high-temperature calcination	Ag/WO₃ nanocomposite	[75]
CVD	The hydroxides of zinc and gadolinium were co-precipitated, then filtered, washed, dried, and calcined	Metal salts, NaOH, etc.	Stir, wash, dry, and calcine	ZnO:Gd nanoparticles	[73]
CVD	The precursor solution of cerium ions reacts with the precipitating agent to precipitate and form CeO_2_ nanoparticles	Metal salts, CTAB, NaOH, etc.	Mix and stir, evaporate and calcine	C-CeO_2_	[77]
CVD	Template preparation, CVD growth	InAs nanowire array, C_2_H_2_, H_2_, etc.	High temperature and high pressure, hydrogen pretreatment, and electron bombardment of Fe catalyst	CNF/InAs hybrid nanostructures	[76]
CVD	Adjust the pH to make Zn_2_^+^ and Lu_3_^+^ react with HCO_3_^−^ for calcination and decomposition.	Metal salts, NaHCO_3_, etc.	Magnetic stirring, PH adjustment, centrifugation, washing, drying, calcination	ZnO/Lu_2_O_3_ nanocomposites	[78]
Template-Assisted Synthesis	Template-guided growth, crystallization, and doping	Metal salts, V_2_O_5_, TEMPO, etc.	High-temperature water heat, filtration, and drying	CoVO-NBs	[83]
Template-Assisted Synthesis	Template-guided growth, no template for comparison	Metal salts, deionized water, ethanol, etc.	Ultrasonic, high-temperature reaction in hydrothermal reactor, ball milling	ZnO-TS, ZnO-TH, ZnO-TM	[82]
Template-Assisted Synthesis	Template-guided growth, cation exchange reaction	Metal salts, TAA, ethylene glycol, etc.	The reaction vessel is heated at high temperature	In_2_S_3_/CuInS_2_	[81]
Ionothermal treatment	Al doping significantly enhances the hydrolysis activity of SrTiO₃	SrTiO_3_, Al_2_O_3_ nanopowder and SrCl_2_	Use air as the atmosphere and conduct for 10 h at 1423 K.	SrTiO_3_:Al	[85]
Photodeposition Method	Photoexcitation and charge separation, deposition of NiO nanoparticles	KNbO_3_ nanorods, CH_4_N_2_S, etc.	Irradiation with 300 W xenon lamp, photodeposition after nitrogen purging, centrifugal washing and drying	NiO/KNbO_3_	[87]

## 4. Modification Strategies for Nanostructured Photocatalysts

In recent years, modification strategies for nanostructured photocatalysts have significantly enhanced their performance through doping engineering, heterostructure construction, and functionalization. For example, directed synthesis of nanowire/column structures using block copolymer self-assembly templates, combined with surface alkylation to enhance the loading of active sites, can achieve a significant increase in mass activity. The composite of bio-inspired photonic crystals and metal-organic frameworks (MOFs) enhances hydrogen evolution reaction (HER) performance, increasing the cathodic current density to 3.64 mA/cm^2^ through synergistic effects of light capture and charge transport. Additionally, heterostructure design (such as 3D porous supports and heterojunction interfaces) can optimize electron transport paths and suppress charge recombination, while atomic-level doping (such as nitrogen or sulfur modifications) can tune the energy band structure to enhance light response. In the future, high-throughput screening and materials genomics will accelerate the development of novel composite catalytic systems, driving the practical application of solar-driven energy conversion technologies.

### 4.1. Doping Engineering

**Figure 8:**
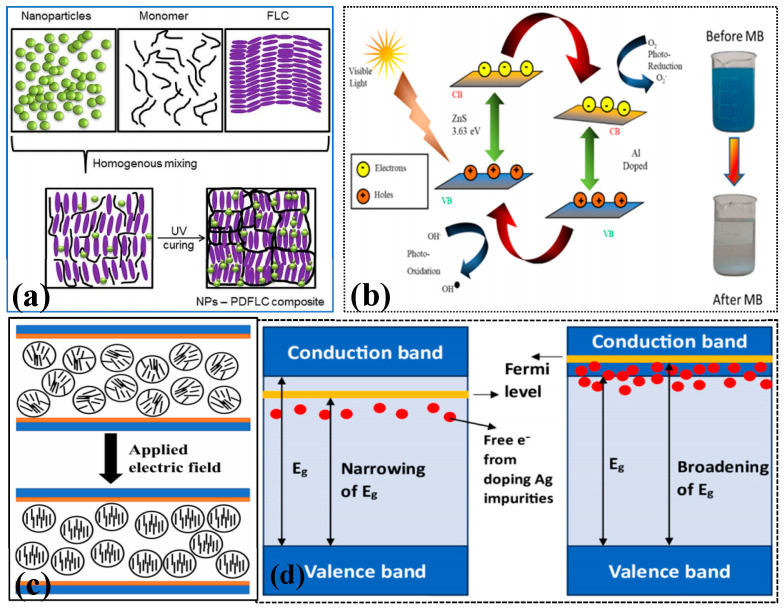
Doping modification of nanostructured photocatalysts (**a**) Reproduced from Ref. [95] with permission from Elsevier, copyright 2025; (**b**) Reproduced from Ref. [96] with permission from Springer Nature, copyright 2025; (**c**) Reproduced from Ref. [100] with permission from MDPI, copyright 2025; (**d**) Reproduced from Ref. [98] with permission from Elsevier, copyright 2025.

Doping can adjust the energy band structure of materials, improving light absorption and carrier separation efficiency. Butt et al. [94] synthesized LaCeO_3_ and B-site doped (Fe/Cr/Zn/Cu) LaCe_1−x_M_x_O_3+δ_ nanocrystals using a coprecipitation method. Nitrates were used as precursors, and ammonia water served as the precipitating agent. After centrifugation, drying, and calcination at 550 °C, the target product was obtained. This process is cost-effective and easy to operate. It is also scalable for the controlled preparation of perovskite-type ceramic materials. As a result, it provides a material foundation for catalytic applications and offers a low-cost alternative to precious metal catalysts. Jayoti et al. [95] discussed the effect of trace amounts of ZnO nanoparticles (0.45–1.0 wt%) doping on the properties of polymer-dispersed ferroelectric liquid crystals (PDFLCs). They found that as the doping amount increased, the dielectric constant and spontaneous polarization enhanced, but the relaxation frequency decreased. The response time improved, and the photoluminescence characteristics were enhanced. These performance changes were attributed to the synergistic regulation mechanism of elastic properties and surface morphology (as shown in Figure 8a). Selvaraj et al. [96] used a sol-gel method to add Na_2_S to a mixed solution of ZnCl_2_ and AlCl_3_. The mixture was continuously stirred for 12 h at pH = 4 and then heated to 200 °C in a muffle furnace to prepare Al-doped ZnS nanoparticles (as shown in Figure 8b). They calculated the energy gap of the material through electro-optical analysis and observed the degradation efficiency of methylene blue (MB). It was found that doping Al into ZnS could enhance the photocatalytic activity of the material. Iqbal et al. [97] synthesized Fe-doped NiO nanoparticles using a coprecipitation method. Ni(NO_3_)_2_·6H_2_O was used as the precursor, which was added to distilled water, stirred, and ultrasonicated. After washing, drying, and calcining, the nanoparticles were obtained. The experimental results showed that the prepared nanomaterials exhibited good charge/discharge retention and excellent optical properties. Veni et al. [98] dissolved ZnCl_2_ and SnCl_2_ in distilled water, then dissolved chitosan into the solution. Different proportions of AgCl were added, and the mixture was stirred for 4 h. After washing, drying, and calcining at 500 °C for 3 h, Ag-doped ZnSnO_3_ nanoparticles were synthesized. The synthesized nanomaterials degraded 95% of CV and 52% of AR dyes within 100 min. As shown in Figure 8d, after doping with Ag, the material’s band gap narrowed, demonstrating its good water remediation and pollutant removal capabilities. Rogolino et al. [99] focused on the photocatalytic synthesis of H_2_O_2_ and found that fully protonated PHI exhibited high activity under visible light, efficiently converted O_2_ to H_2_O_2_, and had a long excited state lifetime. They also pointed out that the introduction of transition metals decreased the performance, which provided a new idea for the application of low-cost materials in the synthesis of photocatalytic H_2_O_2_. Transition metal ions, such as iron (Fe), cobalt (Co), nickel (Ni), etc.; rare earth metal ions, such as common cerium (Ce), lanthanum (La), etc.; and non-metallic ion dopants, such as N and S, etc., can be used to improve the activity and stability of the catalyst through different effects.

### 4.2. Construction of Heterojunctions

**Figure 9:**
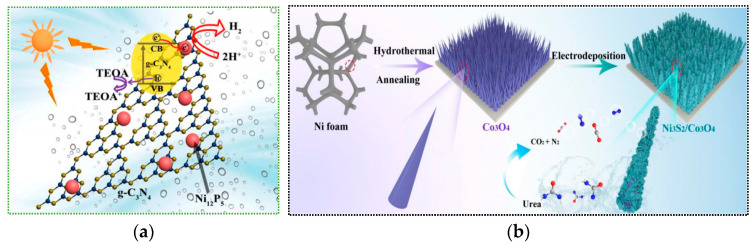
Modification of heterojunctions constructed by nanostructured photocatalysts (**a**) Reproduced from Ref. [102] with permission from Chemistry Select, copyright 2025; (**b**) Reproduced from Ref. [103] with permission from Springer Nature, copyright 2025.

The construction of heterojunctions optimizes the separation and transfer of photogenerated charge carriers through interface band engineering, which is a key strategy to improve photocatalytic efficiency. The principle is based on the band alignment of different semiconductor materials (such as p-n type, Schottky junctions, or Z-scheme heterojunctions). Band bending creates an internal electric field that drives the efficient separation of e^−^-h^+^ pairs and suppresses recombination. Huang et al. [101] synthesized branched Ag-ZnO heterojunction nanostructures using a one-pot method, using (Zn(NO_3_)_2_·6H_2_O and AgNO_3_) as precursors and diethanolamine as a structural directing agent. The reaction was carried out at 180 °C for 12 h to successfully prepare the branched Ag-ZnO heterojunction. The branched Ag-ZnO heterojunction’s unique morphology helps enhance photogenerated charge carrier separation and surface reaction activity, providing important insights for the synthesis and application of other branched metal/semiconductor heterojunction nanostructures. Qin et al. [102] used ultrasound-assisted mixing to combine Ni_12_P_5_ nanoparticles with porous g-C_3_N_4_ in a DMF/hexane/ethanol solution, followed by centrifugation and vacuum drying at 120 °C for 6 h to construct a tightly bound heterojunction (as shown in Figure 9a). This resulted in a visible-light-driven hydrogen production activity of 535.7 µmol g^−1^·h^−1^ (AQY = 4.67%). The excellent performance is attributed to efficient charge separation, providing a new approach for the design of non-precious metal photocatalysts. Yi et al. [103] electroplated Ni_3_S_2_ nanosheets onto a Co_3_O_4_-NF substrate using cyclic voltammetry, washed the product with water, and then vacuum-dried it at 60 °C for 12 h to obtain Ni_3_S_2_/Co_3_O_4_. This was used to construct a foam nickel-supported Ni_3_S_2_/Co_3_O_4_ p-n heterojunction catalyst (as shown in Figure 9b). The built-in electric field at the interface creates electrophilic/nucleophilic regions, promoting urea adsorption and decomposition. The catalyst achieved 10 mA cm^−2^ at only 1.288 V and operated stably for 100 h, providing a new approach for the design of efficient UOR catalysts. Liu et al. [104] constructed an Ag-Ag_2_Se@CdSe double Z-scheme heterojunction with TiO_2_ as the support using multiple methods. The synergistic plasmonic effect enhanced charge carrier separation, achieving 99% degradation of tetracycline (within 120 min), 51.64 μmol·g^−1^·h^−1^ hydrogen production, and efficient antibacterial performance. Tong et al. [105] developed an ionic carbon-nitride catalyst, 2%Ox-KPHI, which achieves an apparent quantum efficiency of 41% at 410 nm through structural distortion and defect site introduction for the highly efficient photocatalytic synthesis of H_2_O_2_ without the need for a co-catalyst. Metal-semiconductor heterojunctions, semiconductor-semiconductor heterojunctions, and oxide-oxide heterojunctions can enhance the stability of catalysts due to their different structures.

### 4.3. Interface Engineering and Surface Modification

**Figure 10:**
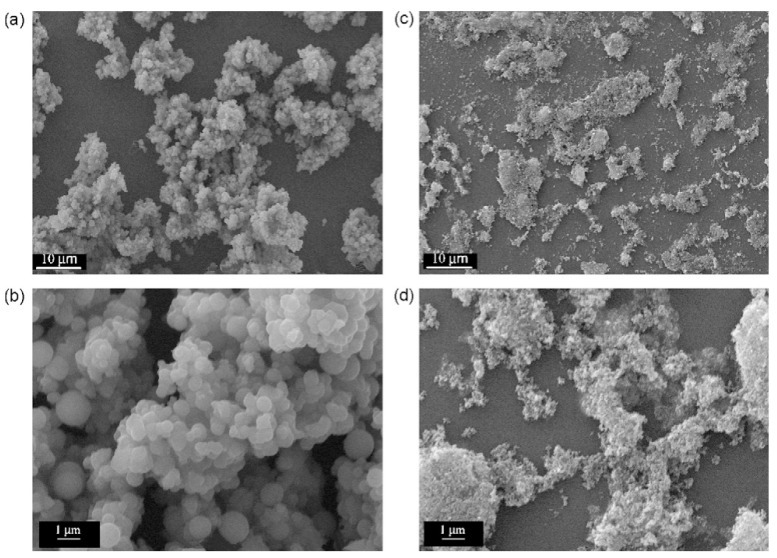
Interface engineering and surface modification of nanostructured photocatalysts Pictures, SEM images of samples SCTE (**a**,**b**) and SCTP (**c**,**d**), at different amplifications, Reproduced from Ref. [106] with permission from Elsevier, copyright 2025.

Surface modification can optimize the interface properties of catalysts, improve charge carrier transport paths, and reduce recombination rates. Through defect engineering or co-catalyst loading, surface modification regulates the surface electronic states, enhances active site exposure, and promotes reactant adsorption. This synergistically improves charge separation efficiency and redox kinetics, providing new directions for environmental remediation while enhancing catalytic activity and stability. Bizarro et al. [106] modified the surface of TiO_2_ films with microspheres by spin-coating to increase the specific surface area. As shown in Figure 10, scanning electron microscope images of samples under different amplification conditions are presented. These images demonstrate that the photocatalytic degradation rate of the modified TiO_2_ films is 62 times higher than that of regular films. Additionally, the modified films have better adhesion and reusability. In this way, the modified TiO_2_ films combine both high efficiency and stability. Zhang et al. [107] prepared Cu-ZSM-5 via ion exchange, followed by two rounds of TEOS chemical liquid-phase deposition (hexane reflux at 90 °C), centrifugation, drying, calcination at 500 °C for 4 h, and then aging in humid air at 750 °C for 13 h to obtain the Cu-ZSM-5-CLD catalyst. An inert silica protective layer was constructed on the surface of Cu-ZSM-5 to inhibit the desorption of Cu^2+^ and zeolite de-alumination during hydrothermal aging, significantly improving the catalytic stability in NH_3_-SCR. Zhou et al. [108] functionalized carbon nanotubes (CNT) using H_2_O_2_ oxidation, introducing phenolic, aldehyde, and other basic functional groups, which increased the active sites. Meanwhile, this oxidation process caused defects in the nanotube walls and surface roughening, helping to improve the styrene yield. Alkhalifa et al. [109] green synthesized ZrO_2_ nanoparticles using Asphodelus fistulosus plant extract as a reducing agent and stabilizer. The material exhibited excellent photocatalytic activity, achieving approximately 92% degradation efficiency of Turkish red dye under Arctic conditions, providing a sustainable and eco-friendly alternative for environmental remediation. Chen et al. [110] investigated the development of NiMoSx-NH_2_ catalysts for 5-HMF oxidation and 4-NP reduction in a pairwise electrocatalytic system to achieve high conversion, yield, and high Faraday efficiency with low cell voltage. Interfacial engineering optimizes the stability of catalysts by optimizing the structure between different phases and facilitating the transfer and transport of electrons between different components. Surface modification can change the chemical composition and structure of the catalyst surface and improve the stability and activity of the catalyst by introducing common atomic groups, such as -OH, -NH_2_, -COOH, and so on.

## 5. The Application of Nanostructured Photocatalysts in the Environmental Field

### 5.1. Water Pollution Treatment

**Figure 11:**
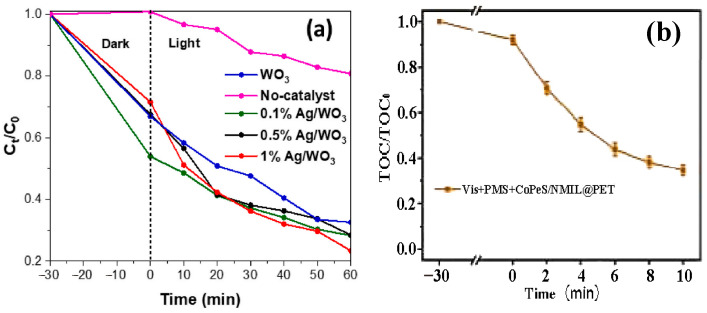
Degradation map of water pollution (**a**,**b**) Reproduced from Refs. [75,112] with permission from Elsevier, copyright 2025.

Nanostructured photocatalysts significantly enhance the degradation efficiency of pollutants through light energy, showing broad application prospects in environmental remediation. In the field of environmental restoration, these catalysts are widely used in water pollution treatment and the efficient degradation or reduction in heavy metal ions (such as Hg^2+^, Ag^+^) to non-toxic substances, while also enabling the recycling of heavy metals. Matalkeh et al. [75] prepared silver nanoparticles on WO_3_ through a deposition-precipitation method. The synthesized catalyst exhibited excellent methylene blue degradation performance (Figure 11a shows the degradation curve of MB, with approximately 80% degradation efficiency) and antibacterial activity, showing significant inhibitory effects against *E. coli* and *S. aureus*. It also demonstrated good stability and reusability. Alpro et al. [34] used algae to green-synthesize metal oxide nanoparticles (MONPs), which offer both environmental friendliness and cost-effectiveness. Compared to plants and fungi, algae are easier to scale up and can efficiently produce multifunctional materials. When combined with photocatalytic technology to degrade industrial wastewater pollutants, they provide advantages such as fast oxidation and low byproduct formation, offering a sustainable strategy for water remediation. Sun et al. [111] developed a photocatalytic self-supported Fenton system based on a 2D/2D Bi_2_Fe_4_O_9_/ZnIn_2_S_4_ van der Waals S-type heterojunction. The system achieved an 88.8% tetracycline degradation rate under visible light, significantly improving photogenerated charge carrier separation and Fe^3+^/Fe^2+^ conversion efficiency and showing potential for formaldehyde degradation. Wu et al. [112] developed a CoPcS/NMIL@PET superhydrophobic nanofiber membrane with self-cleaning and multifunctional properties. The membrane achieved over 99% oil-water detergent separation efficiency under gravity filtration and enabled efficient self-cleaning and formaldehyde degradation under visible light. It also showed a high TOC removal rate (as shown in Figure 11b) and exhibited excellent stability and reusability. Xue et al. [113] developed a photo-Fenton synergistic system by growing FeOOH nanoneedles on the surface of biochar using a two-step carbonization-hydrothermal method. Under visible light, the system degraded 92% of tetracycline in 90 min (H_2_O_2_ promotes the Fe^3+^/Fe^2+^ cycle), with ·OH and h^+^ as the active species. The system also demonstrated excellent magnetic recoverability, providing a new strategy for the treatment of antibiotic wastewater. Ullah et al. [35] synthesized Co_3_O_4_/ZnO heterojunctions through co-precipitation of chitosan/PVP, enhancing the specific surface area and suppressing charge recombination. The 4% CS system achieved 83.33% degradation of RhB under visible light and a 7.75 mm antibacterial zone. Molecular docking revealed that the antibacterial mechanism is mediated by inhibiting TyrRS and DNA gyrase.

### 5.2. Air Pollution Control

**Figure 12:**
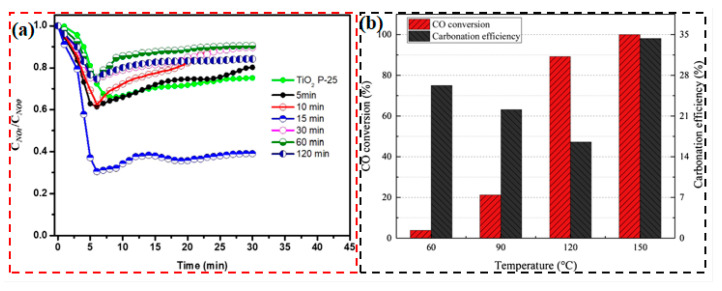
Air pollution control (**a**,**b**) Reproduced from Refs. [114,115] with permission from Elsevier, copyright 2025.

Nanostructured photocatalysts have demonstrated significant advantages in air pollution treatment by virtue of their efficient light-driven mechanism. The core principle is to enhance the redox reaction activity through the excitation and migration of photogenerated carriers to realize the efficient degradation of pollutants. Adriana et al. [114] synthesized BiOI nanomaterials using a microwave solvothermal method. To illustrate the impact of different structures on the photocatalytic performance, Figure 12a shows the degree of NO photocatalytic oxidation conversion for nanomaterials with various structures. The BiOI nanomaterial sample demonstrated superior NO photocatalytic activity under visible light compared to TiO_2_ P-25 within 15 min. When applied to plaster building materials, it showed potential for air purification, providing a new strategy for commercial NO removal. Lin et al. [115] prepared copper manganese cerium/KOH composite catalysts through wet impregnation, solid-state impregnation, and a combined method. The wet/solid-state impregnated CuMnCe/KOH-WSI efficiently oxidized CO and captured CO_2_ in situ at 150 °C due to the large KOH phase. Figure 12b shows the CO conversion rate and carbonization efficiency, while samples with high dispersed KOH exhibited inhibited catalytic performance due to pore blockage. This provides an optimized strategy for air purification in enclosed spaces. Le Pivert et al. [116] directly grew ZnO nanostructures on building materials using a hydrothermal method to develop environmentally friendly photocatalytic functional materials. Large-scale simulation tests showed that these materials could simultaneously degrade pollutants such as O_3_ and NO_x_ in automobile exhaust. The study also revealed that asphalt surfaces may release pollutants and interfere with the Chapman cycle under light exposure. This provides a new perspective for urban air purification and the design of green building materials. Bhandari et al. [117] developed Co_3_O_4_@ZrO_2_ nanocatalysts for the direct oxidation of cyclohexane to adipic acid using air as the oxidant under solvent- and initiator-free conditions. The catalyst achieved a conversion rate of 40% and selectivity of 43%. The acidic sites (1.08 mmol/g) and high Co_3_O_4_ loading enhanced the activity, and the catalyst remained stable over 5 cycles, providing an efficient strategy for the green synthesis of adipic acid. Chausali et al. [37] optimized charge carrier transport pathways by constructing heterojunctions and combining them with defect engineering or co-catalyst loading to enhance surface active sites, thereby synergistically improving charge separation efficiency and reaction kinetics, providing an innovative direction for environmental remediation. Xia et al. [118] constructed a Zn/S dual-vacancy ZnIn_2_S_4_ catalyst, which activates N_2_ and H_2_O synergistically through vacancies. Photo-generated electrons are confined at S vacancies and migrate through In^3+^ to reduce N_2_ to produce NH_3_. Zn/S vacancies serve as active sites to generate OH/O_2_^−^ achieving efficient photocatalytic nitrogen reduction and hexachlorobenzene degradation synergistically.

### 5.3. Carbon Dioxide Reduction and Resource Utilization

**Figure 13:**
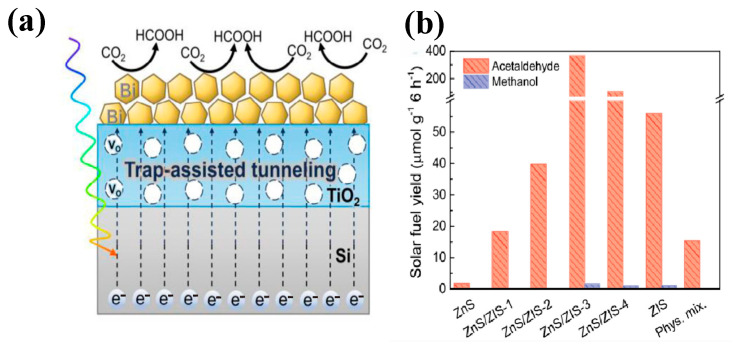
Carbon dioxide reduction field (**a**) Reproduced from Ref. [119] with permission from NANO MICRO Small, copyright 2025; (**b**) Reproduced from Ref. [29] with permission from Elsevier, copyright 2025.

Nanostructured photocatalysts are emerging in the field of carbon dioxide reduction and resource utilization. Specifically, their unique nanoscale effects significantly increase the number of active sites and greatly enhance catalytic performance. Xing et al. [119] constructed a TiO_2_ interlayer with oxygen vacancies in the Si/dT/Bi photoelectrode to accelerate charge carrier transport and induce the growth of Bi sponge-like nanostructures. This enhanced the CO_2_ adsorption active site density and promoted the formation of the key intermediate *OCHO, improving formic acid selectivity and activity. The mechanism was revealed using in situ infrared spectroscopy. Figure 13a illustrates the process of reducing CO_2_ to HCOOH, providing a new strategy for efficient CO_2_ reduction. He et al. [120] assembled a TiO_2_/Cu_2_O heterojunction using a microdroplet method and in situ grew Cu_3_(BTC)_2_ MOF to construct a ternary nanocomposite. The heterojunction synergistically coordinated with unsaturated Cu sites, enhancing charge density and CO_2_ activation, achieving efficient photocatalytic CO_2_ reduction with a preference for CH_4_ generation. The reaction mechanism was revealed using in situ infrared spectroscopy. Sabbah et al. [29] constructed a ZnS/ZnIn_2_S_4_ direct Z-scheme heterojunction in one step via a hydrothermal method. The interface microstrain induced an internal electric field, promoting charge separation. At a Zn:In ratio of 1:0.5, they achieved a 0.8% photocatalytic quantum efficiency (200 times higher than pure ZnS). Spectroscopic and infrared characterization revealed the CO_2_ adsorption and reaction pathways. Figure 13b compares the fuel generation rate of the composite material after 6 h under visible light, providing a new strategy for designing efficient CO_2_ reduction photocatalysts. Yang et al. [39] addressed the CO_2_ crisis through material innovation, including photoelectrodes and nanocarriers. They utilized photoenzymatic, electroenzymatic, and photoelectroenzymatic catalytic systems to achieve the directed conversion of CO_2_ into high-value chemicals, breaking through the limitations of traditional biological carbon fixation. Focusing on coupling mechanisms, material properties, and bottlenecks, they proposed strategies to enhance energy transfer and optimize stability, providing a new paradigm for efficient carbon fixation. Thus, this not only offers a powerful tool for mitigating the greenhouse effect but also opens new pathways for the recycling of carbon resources, bringing new hope for the collaborative development of sustainable energy and chemical production.

### 5.4. Antibacterial and Biological Pollution Control

**Figure 14:**
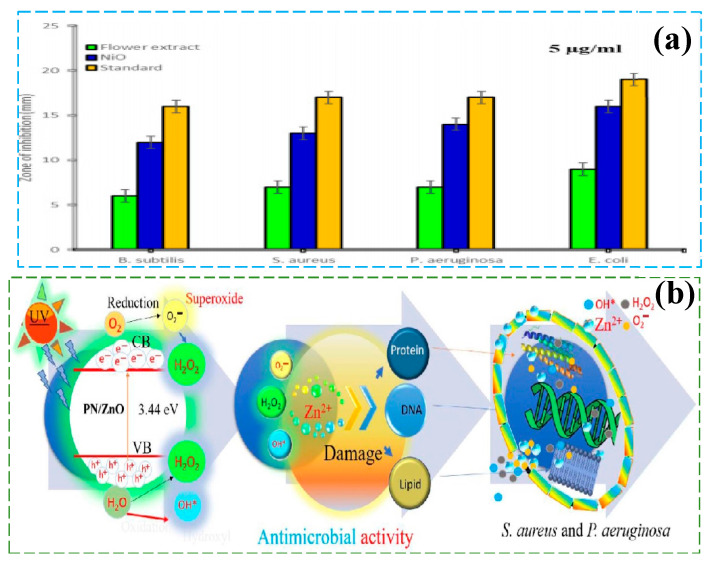
In the field of antimicrobial and biological contamination, (**a**) Reproduced from Ref. [121] with permission from Springer Nature, copyright 2025; (**b**) Reproduced from Ref. [123] with permission from IOP Science, copyright 2025.

Nanostructured photocatalysts rely on the synergistic effect induced by photoexcitation and show unique advantages in the field of antimicrobial and biological pollution treatment. Their mechanism of action primarily relies on photogenerated charge carriers (e^−^-h^+^) that induce the generation of reactive oxygen species (ROS, such as ·OH, O_2_^−^, and H_2_O_2_), which damage microbial cell membranes, proteins, and genetic material. For example, silver-doped TiO_2_ nanoparticles can efficiently kill multidrug-resistant bacteria (such as Escherichia coli and Staphylococcus aureus) under visible light, with antibacterial efficiency 3–5 times higher than traditional photocatalysts. They also reduce the risk of secondary pollution by inhibiting biofilm formation. Additionally, zinc oxide/graphene heterojunction materials enhance light absorption and interfacial charge transfer, achieving a 99.9% bacterial inactivation rate under low-intensity light and effectively degrading antibiotic residues in water. Al-Zaqri et al. [121] used senna leaf extract for the green synthesis of NiO nanoparticles, which exhibited excellent antibacterial performance (Figure 14a shows the schematic of the antimicrobial activity of NiO nanoparticles) and photocatalytic MB degradation activity. The degradation rate reached 97% within 90 min, showing potential for environmental pollution remediation and human health protection applications. Amiri et al. [122] focused on the latest advances and challenges of TiO_2_ nanoparticles and their nanocomposites in antibacterial, antifungal, antiviral, and photocatalytic applications. Due to their stability, non-toxicity, and low cost, TiO_2_ is considered an ideal antibacterial photocatalytic material with broad potential for medical and industrial applications. Gaur et al. [123] studied the green synthesis of ZnO nanoparticles using Piper nigrum plant extract and systematically analyzed their structure and multifunctional properties. The PN/ZnO nanoparticles exhibited excellent catalytic performance, achieving a dye degradation rate of 94.72% for RY-17, and also demonstrated good antibacterial activity. Moreover, Figure 14b illustrates the antibacterial mechanism of PN/ZnO NPs. Therefore, PN/ZnO has broad potential for environmental and pharmaceutical applications. Li et al. [124] developed a novel photocatalytic in situ self-Fenton catalyst RF/EA-Fe@TiC, which achieved a 92% removal rate of tetracycline hydrochloride (TC) within 80 min under visible light. This catalyst not only exhibited high degradation efficiency but also significantly reduced the generation of toxic intermediate products, effectively lowering ecological toxicity. There has been considerable and effective research on the use of nanostructured electro-photocatalysts in antimicrobial and biological pollution control. However, several challenges remain, particularly regarding catalytic activity decay and long-term stability in complex environments. Future research should focus on multi-mechanism synergistic designs (such as combining ultrasound or enzyme catalysis), developing biocompatible coatings to reduce the toxicity of nanomaterials, and exploring their large-scale applications in scenarios like antimicrobial surfaces on medical devices and aquaculture water purification. These efforts will provide innovative solutions to address global challenges. Table 2 summarizes the applications and performance indicators of different materials in the environmental field.

## 6. Conclusions and Prospect

In summary, significant progress has been made in the field of environmental governance with nanostructured photocatalysts. Through the precise regulation of material size, morphology, band structure, and interface properties, researchers have developed highly efficient catalytic systems such as multi-dimensional composites, heterojunctions, and defect engineering, greatly enhancing the separation efficiency of photo-generated charge carriers and light absorption capacity. These advanced catalysts have demonstrated excellent performance in applications such as organic pollutant degradation, water splitting for hydrogen production, and CO_2_ reduction. For example, by constructing 0D/2D or 1D/3D composite structures, the reaction interface is expanded and electron transport is accelerated. At the same time, doping and surface modification strategies effectively reduce carrier recombination rates, improving overall catalytic activity and reaction rates. Furthermore, the introduction of intelligent design and high-throughput screening technologies has made the catalyst optimization process more precise and efficient.

In the future, the development of nanostructured photocatalysts will focus on the following key areas: First, although nanostructured photocatalysts are widely used in environmental treatment, their low quantum efficiency and insufficient long-term stability are still prominent. To address this issue, researchers can explore novel green synthesis and self-healing strategies to improve the durability and stability of catalysts. On the one hand, the light absorption efficiency can be improved by optimizing the energy band structure of the catalyst to better match the solar spectrum. For example, the forbidden bandwidth of the catalyst can be adjusted by elemental doping and constructing heterojunctions to broaden its light response range. On the other hand, the separation and transport efficiency of photogenerated carriers can be improved by improving the surface structure and interfacial properties of the catalyst. For example, designing nanostructured catalysts with specific morphology and surface defects increases the number of their active sites and reduces the carrier complexation probability. Second, the rapid development of nanostructured electro-photocatalysts at the intersection of materials science, chemistry, physics, and information technology will continue. In the future, interdisciplinary integration, through artificial intelligence, data mining, and theoretical calculations, will help deeply analyze the structure-performance relationship and guide the precise design of catalysts. Thirdly, the transformation of nanostructured electro-optical catalysts from laboratory preparation to industrialized large-scale production still faces many challenges. In order to realize the industrial application of catalysts, the following key issues need to be addressed: (1) Cost control: Reducing the preparation cost of catalysts is the first task for industrialization. The production cost can be reduced by optimizing the preparation process, selecting inexpensive raw materials, and developing efficient synthesis methods. For example, the use of low-cost preparation techniques such as hydrothermal and sol-gel methods, as well as the use of renewable resources as raw materials, can help reduce the production cost of catalysts. (2) Stability enhancement: It is crucial to improve the stability of catalysts under actual working conditions. The anti-deactivation ability of the catalyst can be improved by constructing a robust carrier structure, enhancing the interaction between the active components and the carrier, and introducing suitable additives. For example, the use of oxide carriers with high specific surface area and good thermal stability, such as titanium dioxide and alumina, can effectively improve the stability and durability of the catalyst. (3) Large-scale preparation: Achieving large-scale preparation of catalysts is a key step to promote their industrial application. It is necessary to develop preparation techniques suitable for large-scale production, such as continuous flow synthesis and spray drying, to ensure the consistency of catalyst quality and performance. Meanwhile, strict quality control standards and testing methods need to be established to ensure that the performance and quality of each batch of catalyst meet the requirements of industrial application. Overall, with the continuous progress of preparation technology, theoretical models, and intelligent optimization methods, nanostructured photocatalysts will open up broader application prospects for environmental remediation in the future. By solving the problems of low quantum efficiency and insufficient stability, as well as breaking through the bottleneck from laboratory to industrialization, nanostructured electro-optical catalysts are expected to play a greater role in practical environmental remediation and provide strong support for sustainable development.

## Figures and Tables

**Table 2:** Applications and performance indicators of different materials in the environmental field, etc.

**Materials**	**Synthesis Method**	Application Direction	Performance Index	Test Conditions (Light Source, Reaction Time, Catalyst Dosage, etc.)	Ref.
Bi_2_Fe_4_O_9_/ZnIn_2_S_4_	Hydrothermal method (HTM)	Antibiotic (TC) degradation	88.8% degradation rate of TC (120 min)	Visible light, 120 min, TC concentration: 50 mg/L	[111]
Ag/WO_3_	Sol-Gel Technique	Degradation of dye (MB)	80% degradation rate of MB (120 min)	Visible light and UV light, 120 min, 1% Ag/WO_3_	[75]
BC/FeOOH	Carbonization-Hydrothermal Method	Antibiotic (TC) degradation	92% degradation rate of TC (90 min)	Visible light, TC concentration: 20 mg/L, pH = 9	[113]
CS/PVP-Co_3_O_4_/ZnO	Low-temperature Coprecipitation Method (LTCP)	Degradation of dye (RhB)	83.3%degradation rate of RhB	Visible light, Neutral medium, 4%–CS/PVP-Co_3_O_4_/ZnO	[35]
Cu-Mn-Ce ternary catalyst	Wet/Solid-state Impregnation Method	Oxidation of CO	50% conversion rate of CO	Heat from 5 °C/min to 150 °C, Nitrogen flow	[115]
ZnSnO_3_ heterogeneous junction	HTM and PM	conversion of N_2_ to NH_3_	the yield of NH_3_ is 389 μmol/(L·g·h)	Ultrasonic vibration, atmosphere	[125]
BiOI nanostructure	Microwave—Assisted Solvothermal Method (MAM)	NO conversion	61% conversion rate of NO	24 WLED light source, 200 mg catalyst	[114]
ZnIn_2_S_4_V_Zn+S_	Solvothermal Method (STM)	conversion of N_2_ to NH_3_	the yield of NH_3_ is 198.71 μmol/(g·h)	Visible light, reaction for 1 h, isotope labeling	[118]
ZnS-ZIS	Hydrothermal Method (HTM)	CO_2_ reduction	the yields of acetaldehyde and methanol are 367.63 μmol/(g·h) and 1.37 μmol/(g·h).	Visible light, reaction for 3 h,	[29]
Si/dT/Bi and Si/T/Bi	Photoelectrod eposition Method (PED)	CO_2_ reduction	faradaic efficiencies of Si/dT/Bi and Si/T/Bi are 82.7% and 45.5%, respectively.	Simulate sunlight, 28 h and 2 h	[119]
TiO_2_/Cu_2_O/Cu_3_(BTC)_2_	Aerosol Method (AM)	CO_2_ reduction	the CO yield of 310 μmol/(g·h) and the CH_4_ yield of μmol/(g·h)	450 W Xe lamp, 7 h	[120]
PN/ZnO NPs	Hydrothermal method (HTM)	Antibacterial performance (*S. aureus*), (*P. aeruginosa*)	the diameters of the antibacterial zones are respectively 2.93 cm and 3.145 cm	6 W ultraviolet lamp, 37 °C, 24 h, Catalyst dosage 10–40 µL	[123]
CuS:Co NPs	Precipitation Method (PM)	Antibacterial performance (*S. aureus*)	diameter of the antibacterial zone: 2.55 cm	Continuous-wave Nd: YAG laser, 6 mg	[126]
NiO nanoparticles	Green Synthesis Method (GSM)	Antibacterial performance (*E. coli*)	diameter of the antibacterial zone: 23 mm	Sunlight, 37 °C, 24–28 h, Catalyst dosage: 0.2 g/L	[121]
